# The Relationship between Social Support, Empathy, Self-Efficacy, and Humanistic Practice Ability among Clinical Nurses in China: A Structural Equation Model

**DOI:** 10.1155/2023/1378278

**Published:** 2023-11-27

**Authors:** Huan Liu, Lin Zhang, Jin Yan, Hui Huang, Qifeng Yi, Liming Peng

**Affiliations:** ^1^Nursing Department, The Third Xiangya Hospital, Central South University, Changsha, Hunan, China; ^2^Nursing Department, Changsha First Hospital, Changsha, Hunan, China

## Abstract

**Aim:**

This study aims to identify the factors that influence humanistic practice ability, validate the relationship among social support, empathy, self-efficacy, and humanistic practice, and provide reference basis for developing intervention measures.

**Background:**

Cultivating humanistic practice ability in clinical nurses is essential for improving the quality of nursing care.

**Methods:**

From February to March 2022, a cross-sectional survey was conducted in top three hospitals in central China. The study used a self-designed questionnaire to ascertain the general characteristics of the participants. The Nurses' Humanistic Practice Ability Scale, Jefferson Empathy Scale, Perceived Social Support Scale, and General Self-Efficacy Scale were used. Data were analyzed using Spearman's correlation and a structural equation model through statistical product and service solutions (SPSS) and analysis of moment structure (AMOS).

**Results:**

A total of 650 clinical nurses were included in this study. The average age was 32.35 ± 8.35 years. The Nurses' Humanistic Practice ability Scales score was 107.49 ± 19.32. Nurses' humanistic practice ability showed a positive correlation with social support (*r* = 0.455), self-efficacy (*r* = 0.369), and empathy (*r* = 0.375) (all *p* < 0.001). Empathy totally mediated the relationship between social support and humanistic practice ability. In addition, self-efficacy and empathy served as sequential mediators in the association.

**Conclusion:**

Social support can influence the humanistic practice ability through self-efficacy. In addition, the higher the level of social support, the higher the level of self-efficacy, which further promotes the improvement of their empathy and eventually leads to stronger humanistic practice ability. Therefore, the corresponding measures to promote the humanistic practice ability of nurses can be formulated from the abovementioned three aspects. *Implications for Nursing Management*. We recommend that hospital administrators provide nurses with more comprehensive social support and develop intervention strategies to enhance nurses' self-efficacy and empathy, which help to improve the nurse's humanistic practice ability.

## 1. Introduction

Nursing is a profession that focuses on caring for the “whole person.” The human being is a complete being, not a conglomeration of separate anatomical parts [1]. Patients not only need to be the recipients of first-class diagnostic and therapeutic techniques but also need spiritual and psychological comfort, therapeutic involvement, and comprehensive services. Building a harmonious nurse-patient relationship requires the combining of humanism, science, and technology in clinical work [2]. The Healthy China 2030 plan states that medical education reform should focus on medical humanities education and foster humanism in medical students to improve public health care in China. However, compared with the rapid pace of economic development, the progress of medical humanistic education and practice has been relatively slow [3]. Working in a resource-constrained environment, nurses currently place more emphasis on nursing skills than on humanistic care. Some nurses focus only on the treatment of diseases and neglect to interact with patients [4], which is not conducive to the development of good nurse-patient relationships and the improvement of patient health [5].

Developing humanistic practice ability in nurses could resolve this issue. Nurses' humanistic practice ability refers to the clinical nurse's ability to combine humanistic knowledge, skills, spirituality, and use of technology to serve patients. It highlights the behavioral part of nursing and is the outward expression of quality [6]. Nurses with a strong humanistic practice can engage in effective clinical practice, provide high-quality humanistic care to patients, demonstrate respect for patients and a love of life [7], and promote patients' physical and psychological recovery [8]. Humanistic practice also has a positive impact on nurses' daily work activities and personal and cultural values [9]. Recent studies have shown that the humanistic practice ability of clinical nursing staff and nursing students in China should be improved [10, 11]. The studies have found that the ability to practice in a humanistic way can be developed quickly through short-term training [6] and that there is an urgent need to identify the potential factors influencing humanistic practice and promote our understanding of humanistic practice, which can provide a theoretical basis on which nurses can develop multifaceted training programs.

Perceived social support refers to an individual's subjective feeling and evaluation of the degree to which he or she is supported by the outside world [12]. The degree of concern of family members and colleagues for nurses is significantly correlated with their level of humanistic practice ability; nurses who receive care and love are able to generate more care and love, which in turn promotes humanistic practice with patients [13]. Social support may affect nurses' humanistic practice ability.

**Hypothesis 1 .**

Perceived social support is significantly associated with humanistic practice ability.

Empathy is the ability to put oneself in another's shoes [14]. It mainly includes perspective taking, compassionate care, and standing in the patient's shoes. The first factor, perspective taking, is the predominant factor and refers to the cognitive aspects of empathy. The second factor, compassionate care, is characterized by a combination of cognitive and affective aspects of empathy and is also considered an essential factor in professional relationships with patients. The third factor, standing in the patient's shoes, is a concept that is inversed to emotional detachment [15]. In the healthcare context, this means the ability of professionals to put themselves in the shoes of patients and families—to understand their emotions, moods, and psychological conditions and to develop effective nursing interventions that lead to a healthy emotional experience for patients [16]. Empathy is the most valuable virtue in patient-centered care and is a key factor in patient adherence, satisfaction, and outcomes, as well as appropriate care behaviors [17]. One cross-sectional study showed that empathy is closely related to the humanistic caring ability of midwifery students and that empathy may influence the humanistic caring ability of nursing students through the direct and indirect effects of emotional intelligence [10]. Research has shown a positive correlation between empathy and social support, indicating that nurses with more social support have a higher level of empathy [18]. Another research also confirms that social support is considered a protective agent for preventing psychological problems in healthcare providers, helping to improve their empathy. The improvement of empathy can improve the quality of life of nurses and enhance their level of care for patients [19]. Therefore, empathy has been suggested as a powerful predictor of humanistic practice ability.

**Hypothesis 2 .**

Empathy is significantly associated with humanistic practice ability.

**Hypothesis 3 .**

Perceived social support indirectly influences humanistic practice ability through empathy.

Self-efficacy is defined as an individual's capability to carry out the necessary actions that yield specific outcomes, serving as a crucial link between knowledge and behavior [20]. Previous research indicates that nursing students who receive strong social support are likely to demonstrate enhanced self-efficacy [21]. In addition, self-efficacy has been shown to be related to empathy, suggesting that strategies aimed at bolstering self-efficacy could also enhance empathic abilities [22]. Further studies reveal that self-efficacy plays a significant moderating role between organizational atmosphere and the capability for humanistic practice [23]. Individuals with high levels of self-efficacy are often characterized by a sense of resilience and pride in their abilities. They are motivated to overcome challenges, thereby influencing their behavior—especially in the demanding settings of healthcare environments [24, 25]. Based on the evidence, the role of self-efficacy in humanistic practice ability should be identified.

**Hypothesis 4 .**

Self-efficacy is significantly associated with empathy.

**Hypothesis 5 .**

Self-efficacy is significantly associated with humanistic practice ability.

**Hypothesis 6 .**

Perceived social support indirectly influences humanistic practice ability through self-efficacy.

**Figure 1:**
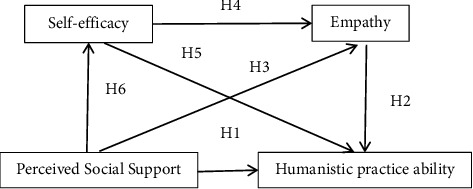
Hypothetical model for factors influencing humanistic practice ability in clinical nurses. H1, hypothesis 1; H2, hypothesis 2; H3, hypothesis 3; H4, hypothesis 4; H5, hypothesis 5; H6, hypothesis 6.

This study will identify factors that influence humanistic practice ability to provide evidence that can be used to further improve nurses' humanistic practice ability and will validate the correlation between clinical nurses' humanistic practice ability and levels of social support, empathy, and self-efficacy. Finally, our study may encourage management to apply interventions to promote humanistic practice for nurses. The hypothesized theoretical model is shown in Figure 1.

## 2. Methods

### 2.1. Setting and Participants

This study involved conducting a cross-sectional survey of nurses in three tertiary hospitals in Changsha. The aim was to identify predictors of clinical nurses' humanistic practice ability, including social support, empathy, and self-efficacy.

#### 2.1.1. Inclusion and Exclusion Criteria

Registered nurses, working in our hospital for at least 1 year, who agreed to participate in the study were included. Exclusion criteria were as follows: advanced practice nurses, intern nurses, and nonclinical nursing positions.

#### 2.1.2. Sample Size Calculation

The questionnaire included three dimensions of comprehension of social support, three dimensions of an empathy scale, and five dimensions of a nurse humanistic practice ability scale and general self-efficacy scale. There were 10 general information factors and a total of 22 statistical analysis variables. The sample size was determined by 10 participants per variable [26]. Based on the sample content estimation formula (max (number of dimensions) *∗* 10) *∗* (100% + 20%), at least 264 participants were needed.

### 2.2. Measures

#### 2.2.1. Participants' General Characteristics

We have designed a questionnaire, which included questions gender, nationality, marital status, age, religion, professional position, total working years, amount of training received, understanding of concepts related to “humanistic care,” and the objective evaluation of their own humanistic knowledge and quality.

#### 2.2.2. Nurses' Humanistic Practice Ability Scale

The scale was developed by Yan et al. to measure the humanistic practice ability of nursing staff in China [6]. The scale contains the following 26 items across five dimensions: humanistic care practice ability (10 items), self-management ability (3 items), interpersonal communication ability (6 items), ethics and legal practice ability (3 items), and psychological adjustment ability (4 items). Each response was converted to a numerical score ranging from 1 to 5, with 1 indicating strongly disagree and 5 indicating strongly agree. Total scores ranged from 26 to 130, with higher scores indicating stronger humanistic practice ability. The tool's reliability had a Cronbach's *α* of 0.979 in the work of Wang et al. [27]. The reliability of our study had a Cronbach's *α* of 0.984.

#### 2.2.3. Jefferson Empathy Scale

This scale, which measures the level of empathic ability of individuals, was developed by Dr. Mohammadreza Hojat et al. [28] and adapted for the Chinese context by Chinese scholars An et al. [29] in 2008. The scale contains the following three dimensions: perspective taking (10 items), compassionate care care (7 items), and walking in patient's shoes (3 items)—a total of 20 items, 10 of which are reverse scored on a 7-point Likert scale, with higher scores associated with greater empathy. The Cronbach's *α* and half coefficients are 0.750 and 0.771, respectively, and the retest reliability is 0.659, which has good reliability in the Chinese population [29]. Cronbach's *α* is 0.896 in the present study.

#### 2.2.4. Perceived Social Support Scale

The scale—a self-assessment scale to measure the individual's perceived level of social support—was developed by Zimet et al. [30] and revised by our scholar Jiang in 2001 [31]. The scale consists of 10 items, with three dimensions—family support (4 entries), friend support (4 entries), and other support (4 entries). All items were rated on a 7-point Likert-type scale, ranging from 1 (strongly disagree) to 7 (strongly agree). The total score range is 12–84. The higher the score, the more social support the individual receives, with 12–36 representing low support, 37–60 intermediate support, and 61–84 a higher level of support. Cronbach's *α* was 0.956 in the present study.

#### 2.2.5. General Self-Efficacy Scale

In this study, the Chinese version of the general self-efficacy scale revised by Wang et al. was used [32] . The scale was derived from the general self-efficacy scale (GSES) developed by German psychologist Schwarzer, which has good reliability. The scale of “disagree,” “not sure,” “agree somewhat,” and “agree strongly” was used, with scores ranging from 1 to 4 and a total score of 10 to 40. Higher scores indicate higher general self-efficacy of nurses. Cronbach's *α* was 0.934 in the present study.

### 2.3. Data Collection

The convenience sampling method was used to select nurses from three tertiary grade A hospitals in Hunan Province as the research object. Data collection was facilitated through an online survey link between February and March 2022. The survey was entered into the Questionnaire Star platform that was developed by Changsha Ranxing Information Technology Co., Ltd, which required an answer for every question to avoid missing data. We organized a presurvey for 10 nurses. We communicated with the hospital nursing department and then explained the purpose and significance of the study to the head nurse. We published recruitment information in the work group, flagged informed consent on the first page of the questionnaire, and highlighted consent before entering the questionnaire to ensure that each participant knew the purpose and content of the study. A total of 665 data were collected online, and during our analysis, we found that 15 of them were duplicate submissions. After deleting duplicates, the effective data were 650 and the questionnaire efficiency was 97.74%. We collected 198, 210, and 242 questionnaires from three hospitals, respectively.

### 2.4. Ethical Considerations

This study was approved by the Hospital Medical Ethics Review Board. All participants participated voluntarily and could withdraw from the study at any time. The survey did not disclose any personal information.

### 2.5. Data Analysis

SPSS version 25.0 (SPSS Inc.) and AMOS version 24.0 (IBM Corp.) were used for statistical analysis. The count data were described by frequency and percentage, and the measurement data were described by the mean ± standard deviation. Spearman correlation analysis was performed to analyze the relationship among social support, empathy, self-efficacy, and humanistic practice ability. A structural equation model was employed to identify both direct and indirect relationships in the model. Standardized and unstandardized path coefficients, variances, *R*-squared values, standard error, and *p* values were reported. Variables with nonsignificant coefficients were removed from the model. Estimates without zero in the 95% confidence interval (CI) indicated that the mediation effects were significant. The measurement model was examined through reliability (Cronbach's *α* and composite reliability), convergent validity, and discriminant validity. The structural model fit was evaluated according to the following standards: *χ*^2^/DF ≤ 5.00, comparative fit index (CFI) ≥ 0.90, incremental fit index (IFI) ≥ 0.90, and standardized root mean square residual (SRMR) ≤ 0.08 [33]. In this study, *α* = 0.05, whereas tests were two tailed.

## 3. Results

### 3.1. Scores of the Humanistic Practice Ability

The average total score of nurses' humanistic practice ability was 107.49 ± 19.32, which was at the upper middle level. The scores for each dimension are shown in Table 1.

### 3.2. General Characteristics

Table 2 shows the general characteristics of the surveyed nurses. A total of 650 clinical nurses were included in this study. The average age was 32.35 ± 8.35 years, 98.3% were female and 1.7% were male, and the average years of nursing experience were 11.02 ± 8.33. 42.9% of the nurses reported that they had never received training related to humanistic practice, 21.1% of nurses reported that they did not understand the concept of humanistic nursing, and 82% of nurses indicated that they lacked humanistic knowledge and qualities.

### 3.3. Measurement Model

The humanistic practice ability of nurses is positively correlated with their understanding of perceived social support (*r* = 0.455), self-efficacy (*r* = 0.369), and empathy (*r* = 0.375) (all *p* < 0.01).  Table 3 shows the correlation between the study variables and the significant correlation between all variables, indicating the legitimacy of establishing a path between them. We used Cronbach's *α* and composite reliability (CR) to test the reliability of the model. All results were greater than 0.7, indicating satisfactory reliability [34]. To test convergent validity, we calculated the average variance extracted (AVE) from each structure to meet the requirement of at least 0.5 in all structures [35].

### 3.4. Structural Model

**Figure 2:**
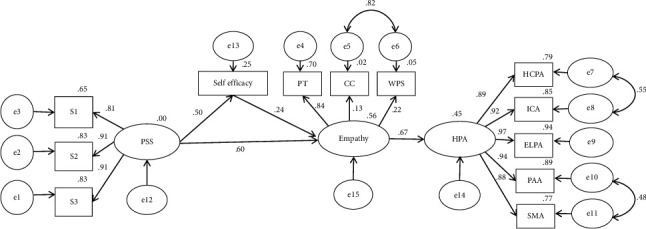
The final model of humanistic practice ability and associated factors (with standardized regression coefficients) (all *p* < 0.05). PSS, perceived social support; S1, family support; S2, friend support; S3, other support. PT, perspective taking; CC, compassionate care; WPS, walking in patient's shoes; HPA, humanistic practice ability; HCPA, humanistic care practice ability; ICA, interpersonal communication ability; ELPA, ethics and legal practice ability; PAA, psychological adjustment ability; SMA, self-management ability.

The standardized path coefficients from social support to perceived humanistic practice ability and self-efficacy to humanistic practice ability are not significant (Table 4). Therefore, we excluded these two paths and reconstructed the mediation model for analysis. The modified model shows an acceptable fit as follows: *χ*^2^/df = 3.784 < 5, RMSEA = 0.065, CFI = 0.982, TLI = 0.976, and GFI = 0.953. The path map and coefficients are shown in Figure 2. Empathy plays a complete mediating role between perceived social support and humanistic practice ability, with a mediating effect value of 0.402. Perceived social support indirectly affects humanistic practice ability through self-efficacy and the complete chain mediating effect of empathy, with mediation effect values of 0.081 (Table 5).

## 4. Discussion

The awareness and ability to provide humanistic care among medical staff are crucial factors affecting patients' psychological states, treatment effectiveness, and overall rehabilitation. Moreover, humanistic care plays a vital role in enhancing the quality of medical services, increasing patient satisfaction, fostering harmonious nurse-patient relationships, and elevating the professional awareness of medical staff [11]. This study found that empathy fully mediated the relationship between perceived social support and humanistic practice ability. In addition, perceived social support can influence the humanistic practice ability through the chain-mediating effect of self-efficacy and empathy. These findings enhance our understanding of the mechanisms connecting these variables and shed light on the factors influencing humanistic practice from both external support and individual perspectives.

In this study, the mean score for nurses' humanistic practice ability was 107.29 (SD = 19.30). This is consistent with the research conducted by Zhang et al. [36]. It may be related to the fact that 57.1% of the nurses in this study have received humanistic nursing related training. Training is effective in promoting humanistic care practices [11, 27]. Among the various dimensions of humanistic practice in this study, interpersonal communication ability was the highest, indicating that clinical nurses have strong communication skills with patients. It is possible that 69.4% of the nurses in this study have worked for more than 5 years, and there are more opportunities for communication among nurses, patients, and medical staff in the long-term clinical nursing work process. The accumulation of professional knowledge and work experience has laid the foundation for improving communication skills. The formation process of humanistic practice ability is a process of long-term accumulation and dynamic learning, which cannot be mastered quickly in a short time. The transformation of ability from quantitative change to qualitative change requires continuous learning and personal perception. Therefore, we need to focus on strengthening the regular training of young and junior nurses to further enhance their humanistic practice ability [36].

The lowest score in the self-management dimension in this study may be related to the high intensity and pressure of clinical nursing work, leading to insufficient emotional management and self-planning among nurses [27]. Nursing managers can improve nurses' resilience through group intervention [37] and reduce nurses' perception of work stress [38] and depression [39] through network-based stress management plans (such as cognitive behavioral techniques), helping nurses master emotional regulation skills and avoid emotional work. At the same time, clinical nurses can receive career planning training. Reasonable self-career planning can help strengthen nurses' awareness of self-directed learning, improve their professional skills, and increase their sense of identification and satisfaction with their work.

The findings of this study reveal that empathy acts as a complete mediator between social support and humanistic practice. This suggests that a supportive work environment fosters empathy among nurses, which, in turn, enhances their capacity for humanistic practice. Notably, higher levels of empathetic competence correlate with stronger humanistic practice abilities. These results are corroborated by a cross-sectional study conducted by Wang et al. [10]. According to Watson's theory of human caring, empathy is identified as one of its ten core components [40]. Within the framework of medical humanism, two essential domains—emotional and cognitive—are emphasized.

Empathy enables us to understand the intentions of others, predict their behavior, and experience emotions triggered by the emotions of others. It can be divided into two parts as follows: the cognitive and affective components [41, 42]. The cognitive part helps us to identify others' emotions at the cognitive level. The emotional part is our ability to feel emotions similar to those of others [43]. Empathy plays a core role in social interaction [44]; people with high empathy ability may more actively understand the care and support of others and will be more deeply involved in the internal state of others, and sympathy for strangers can even produce alternative perception of social support and establish feelings to connect with social network [45].

People with strong empathy are sensitive to the care and support from the outside world. Instead, people with low empathy may not care about the support and care others give them. This may further influence their attitudes towards others. People with high empathy are more likely to perceive support and then show a higher tendency for prosocial behavior [46]. When a person can perceive the existence of social support, he/she will feel a sense of belonging, thus increasing the ability to identify self-worth, have a better understanding of professional responsibilities and obligations, and have the courage and patience to alleviate the pain caused by disease, thus improving their humanistic care ability [47].

Thus, the cultivation of empathy is instrumental in advancing humanistic practice. Existing literature suggests that empathy can be taught and acquired [48]. Various methodologies can be employed by administrators to improve nurses' empathic abilities and, consequently, their competence in humanistic practice. These may include measures to enhance psychological well-being [49], role-playing exercises that facilitate a deeper understanding of patients' perspectives [50], the integration of arts and humanities into the medical curriculum [51], and the use of virtual reality as a pedagogical tool to stimulate visual and emotional learning [52].

Another approach reveals that self-efficacy and empathy have a significant chain-mediating effect between social support and humanistic practice. Nurses with high self-efficacy are more confident and willing to employ empathy in their work, thus facilitating stronger nurse-patient relationships. Improving self-efficacy can serve as a strategy for cultivating empathy [22].

High self-efficacy enables nurses to patiently analyze and accurately perceive problems, particularly in the face of difficulties or setbacks. This confidence influences their level of motivation, which in turn affects their behavior [53]. Moreover, nurses with high self-efficacy believe they possess the skills necessary for effective symptom management, thus influencing the quality of care [54]. Increased self-efficacy encourages nurses to engage more actively in their work, ultimately enhancing patient care and nurse-patient relationships [23]. Research has indicated that self-efficacy mediates the organizational climate in nursing settings and affects humanistic practice ability [23]. In addition, being cared for and loved enhances the caregiver's capacity for care and love, reinforcing their concern for patients [13]. External support from family members and colleagues contributes positively to nurses' personal development, enabling them to better grasp the essence of care giving [55]. Humanistic practice abilities are not innate but are developed over time through experience and perception. As such, managers should provide external support to improve nurses' self-efficacy, cultivate their empathy, and consequently enhance their humanistic professional skills.

In summary, interventions aimed at improving social support, empathy, and self-efficacy may be beneficial for enhancing the humanistic practice capabilities of nurses.

## 5. Limitations and Recommendations

This study is a cross-sectional study, measured and analyzed at a particular time and in a particular place, which may introduce reaction bias and affect the accuracy of causality. In future research, the sample size could be expanded and the causality between variables could be inferred by using a multicenter, longitudinal research method.

## 6. Conclusion

Empathy completely regulates the relationship between perceived social support and human practice ability; moreover, perceived social support can influence human practice ability through the interlocking mediation effects of self-efficacy and empathy. Interventions should focus on improving social support, self-efficacy, and empathy, thus directly or indirectly influencing nurses' humanistic practice abilities.

## 7. Implications for Nursing Management

The results of this study have practical significance in that they could help clinical nurses improve their humanistic practice ability and promote clinical humanistic nursing more broadly. The results emphasized that strategies to improve nurses' humanistic practice ability should focus on improving nurses' empathy, social support, and self-efficacy. Nursing managers could hire professionals to conduct systematic and continuous humanistic training for clinical nurses to improve empathy and self-efficacy. On the other hand, we also should build a harmonious nursing atmosphere to provide nurses with good social support.

## Figures and Tables

**Table 1:** Total score of the nurses' humanistic practice and all dimensions (*N* = 650).

Variables	Minimum	Maximum	Score (mean ± SD)	Average score (mean ± SD)
Humanistic care practical ability	10	50	40.84 ± 7.70	4.08 ± 0.77
Interpersonal communication ability	6	30	25.66 ± 4.72	4.28 ± 0.79
Ethics and legal practical ability	3	15	12.58 ± 2.44	4.19 ± 0.81
Psychology adjustment ability	4	20	16.38 ± 3.13	4.10 ± 0.78
Self-management ability	3	15	12.04 ± 2.38	4.01 ± 0.79
Humanistic practice ability	26	130	107.49 ± 19.32	4.13 ± 0.74

**Table 2:** Participants' characteristics (*N* = 650).

Characteristics	Categories	*N*	(%)
Gender	Male	11	1.7
	Female	639	98.3

Nationality	China's main nationality	614	94.5
	Minority nationality	36	5.5

Marital status	Single	192	29.5
	Married	443	68.2
	Divorced	15	2.3

Age (years)	≤25	131	20.2
	26–30	193	29.6
	31–35	156	24.0
	36–40	79	12.2
	≥40	91	14.0

Religion	Yes	31	4.8
	No	619	95.2

Professional position	Nurse	133	20.5
	Primary nurse	239	36.7
	Nurse-in-charge	231	35.5
	Cochief superintendent nurse	44	6.8
	Chief superintendent nurse	3	0.5

Total working years	<5	199	30.6
	5–15	293	45.1
	16–25	104	16.0
	>25	54	8.3

Amount of training received	Not accepted	279	42.9
	<1 time/year	98	15.1
	1 time/year	117	18.0
	2-3 times/year	91	14.0
	>3 times/year	65	10.0

Understanding of concepts related to “humanistic care”	Very well understood	111	17.1
	General understanding	402	61.8
	Unclear	59	9.1
	Some understanding	65	10.0
	No understanding at all	13	2.0

The objective evaluation of their own humanistic knowledge and quality	Very deficient	38	5.8
	Comparatively lacking	495	76.2
	Not lacking	117	18.0

**Table 3:** Descriptive statistics and correlations among study variables.

Variables	Mean (SD)	Standardized factor loading	Cronbach's *α*	CR	AVE	1	2	3
(1) Perceived social support	67.56 (11.51)	0.726–0.921	0.956	0.966	0.704			
(2) Self-efficacy	26.78 (6.25)	0.555–0.882	0.934	0.933	0.585	0.478^*∗∗*^		
(3) Empathy	113.01 (16.84)	0.509–0.886	0.896	0.961	0.555	0.382^*∗∗*^	0.106^*∗∗*^	
(4) Humanistic practice ability	107.50 (19.32)	0.528–0.941	0.984	0.988	0.762	0.455^*∗∗*^	0.369^*∗∗*^	0.375^*∗∗*^

AVE, average variance extracted; CR, composite reliability; SD, standard deviation. ^*∗∗*^*p* < 0.01.

**Table 4:** Model path coefficient.

Endogenous variables	Predicting variables	*R* ^2^	*B*	*B*′	SEs	*t*	*p*value	Lower	Upper
Self-efficacy	Perceived social support	0.247	0.826	0.497	0.061	13.467	^*∗∗∗*^	0.434	0.554

Empathy	Perceived social support	0.591	1.058	0.629	0.080	13.274	^*∗∗∗*^	0.420	0.799
	Self-efficacy		0.232	0.229	0.045	5.169	^*∗∗∗*^	0.122	0.346

Humanistic practice ability	Perceived social support	0.482	−0.175	−0.096	0.344	−0.509	0.610	−1.585	0.210
	Self-efficacy		0.025	0.023	0.086	0.292	0.770	−0.533	0.152
	Empathy		0.810	0.751	0.317	2.558	0.011	0.355	2.605

^*∗∗∗*^ *p* < 0.001.

**Table 5:** Results of mediation effect analysis.

Mediation effects	Estimated effects	SE	Bootstrapping 95% CI		*p* value
			Lower	Upper	
Perceived social support ⟶ empathy ⟶ humanistic practice ability	0.402	0.048	0.306	0.492	0.001
Perceived social support ⟶ self-efficacy ⟶ empathy ⟶ humanistic practice ability	0.081	0.018	0.048	0.120	0.001

## Data Availability

The data used to support the findings of this study are available on request from the corresponding authors.
